# Association between different diet quality scores and depression risk: the REGICOR population-based cohort study

**DOI:** 10.1007/s00394-024-03466-z

**Published:** 2024-08-24

**Authors:** Gabriela Lugon, Álvaro Hernáez, Felice N Jacka, Jaume Marrugat, Rafael Ramos, Josep Garre-Olmo, Roberto Elosua, Camille Lassale

**Affiliations:** 1 Epidemiology and Public Health Programme, Hospital del Mar Medical Research Institute, PRBB, Carrer Doctor Aiguader 88, Barcelona, 08003 Spain; 2 Preventive Medicine and Public Health Training Unit PSMar-UPF-ASPB (Parc de Salut Mar - Pompeu Fabra University - Agència de Salut Pública de Barcelona), Barcelona, Spain; 3 PhD Program in Biomedicine, Universitat Pompeu Fabra (UPF), Barcelona, Spain; 4 Consortium for Biomedical Research - Pathophysiology of Obesity and Nutrition (CIBEROBN), Instituto de Salud Carlos III, Madrid, Spain; 5 Centre for Fertility and Health, Norwegian Institute of Public Health, Oslo, Norway; 6 Facultat de Ciènces de la Salut Blanquerna, Universitat Ramon Llull, Barcelona, Spain; 7 School of Medicine, Food & Mood Centre, IMPACT Strategic Research Centre, Deakin University, Melbourne, VIC Australia; 8 Consortium for Biomedical Research - Cardiovascular Diseases (CIBERCV), Instituto de Salud Carlos III, Madrid, Spain; 9 Institut Universitari d’Investigació en Atenció Primària Jordi Gol (IDIAP Jordi Gol), Girona, Spain; 10 Girona Biomedical Research Institute (IdIBGi), Dr. Josep Trueta University Hospital, Girona, Spain; 11 Department of Medical Sciences, School of Medicine, University of Girona, Girona, Spain; 12 Serra-Húnter Professor Department of Nursing, University of Girona, Girona, Spain; 13 Faculty of Medicine, University of Vic - Central University of Catalunya, Vic, Spain; 14 ISGlobal, Barcelona, Spain; 15 Universitat Pompeu Fabra (UPF), Barcelona, Spain

**Keywords:** Depression, Dietary score, Prevention, Nutritional psychiatry

## Abstract

**Background:**

Our aim was to determine the association between diet quality and depression incidence in the population-based REGICOR cohort study, Catalonia, Spain.

**Methods:**

Prospective observational study using participants’ baseline (2003–2006), follow-up (2007–2013) and clinical records data. Five diet quality scores were derived from a food frequency questionnaire (FFQ) at baseline: the relative Mediterranean Diet Score (rMED), the Modified Mediterranean Diet Score (ModMDS), a Dietary Approaches to Stop Hypertension (DASH) score, a Healthful Plant-based Diet Index (HPDI) and the World Health Organization Healthy Diet Indicator (WHO-HDI). Participants using pharmacological antidepressant treatment were excluded as a proxy for presence of depression at baseline. At follow-up, the Patient Health Questionnaire (PHQ-9) was applied to assess depressive symptoms (≥ 10 defining depressive disorder). A secondary outcome was depression diagnosis assessed through clinical records. Logistic regression and Cox proportional hazards models were used.

**Results:**

Main analysis included 3046 adults (50.3% women) with a mean age of 54.7 (SD = 11.6) years. After 6-years follow-up, 184 (6.04%) cases of depressive disorder were identified. There was 16% lower odds of depressive disorder per 1SD increase of rMED (OR = 0.84; 95%CI = 0.71–0.98). Secondary outcome analysis (*n* = 4789) identified 261 (5.45%) incident cases of clinical depression diagnosis over 12 years follow-up, and 19% lower risk of clinical depression was observed with the WHO-HDI (HR = 0.81; 95%CI = 0.70–0.93). Adjusting for BMI did not attenuate the findings.

**Conclusions:**

A significant inverse association between diet quality and depression incidence was found in this population-based cohort study, independent of sociodemographic, health and lifestyle. Adherence to a healthy diet could be a complementary intervention for the prevention of depression.

**Supplementary Information:**

The online version contains supplementary material available at 10.1007/s00394-024-03466-z.

## Introduction

According to the 2019 Global Burden of Disease Study, 5% of adults (280 million people worldwide) suffer from depression, making depressive disorders the second leading cause of years lived with disability in the world [1]. Prevalence has been reported to be slightly higher in European regions such as Catalonia (Spain) (7.2% in 2019, 10.6% in 2020), particularly in women (13.7%), individuals 75 years of age or older (18.8%), the less favored social class (14%) and those with a lower educational level (15.2%) [2].

Depression is a multifactorial disorder [3] with high probability of relapses [4], for which standard treatments are not always available or effective [4, 5]. Therefore, efforts have been recently made to uncover new modifiable risk factors, in particular diet and physical activity, smoking and sleep [6, 7]. There are different biological pathways through which an inadequate diet could influence depression, including increased levels of inflammation, oxidative stress, the microbiota-gut-brain axis, and disturbances to the hypothalamus-pituitary-adrenal system with overproduction of cortisol, among others [8]. Obesity and depression are connected in a bi-directional relationship [9] and diet is a driver of body weight and thus on the causal pathway; it is therefore important to study the diet-depression association independently of adiposity [10, 11].

In the last decade, there has been an increasing number of studies revealing relationships between higher diet quality and lower risk of depression, but the majority are cross-sectional studies in which causality cannot be determined [12]. We conducted a meta-analysis of prospective studies in 2018 and found that the risk of depression was lower in people with a high Mediterranean diet adherence score [12]. When updating this systematic review until 2022, we found that among the newly published prospective studies, various report a significant inverse association between a higher diet quality and the risk of depression [13–17], with the most frequently studied dietary pattern being a traditional Mediterranean diet [18–21]. However, it should be noted that some of these studies include volunteers with specific characteristics such as only older adults, university graduates, male workers, or only women, and that population-based studies are lacking to extrapolate results to the general population.

The main objective of this study was to determine the association between diet quality and the incidence of depression in a population-based cohort of adults living in the Girona province (Spain). Secondary objectives were: (1) to compare the association with depression with different diet quality scores; and (2) to assess the mediating effect of body weight.

## Methods

### Study population

The “Registre Gironí del Cor” (REGICOR) study is a prospective population-based cohort of free-living adults aged 35–79 years carried out in the province of Girona (Spain) with the aim of studying the magnitude of cardiovascular diseases and their determinants in Catalonian general population. Three baseline cohorts were recruited in 1994–1996 (*n* = 1,748), 1999–2001 (*n* = 3,058), and 2003–2006 (*n* = 6,352). All these participants were invited to attend a second visit in 2007–2013 and have a clinical follow-up with linkage to electronic health records until 31 December 2016. Further details have been described previously [22].

### Design and participants

**Fig. 1:**
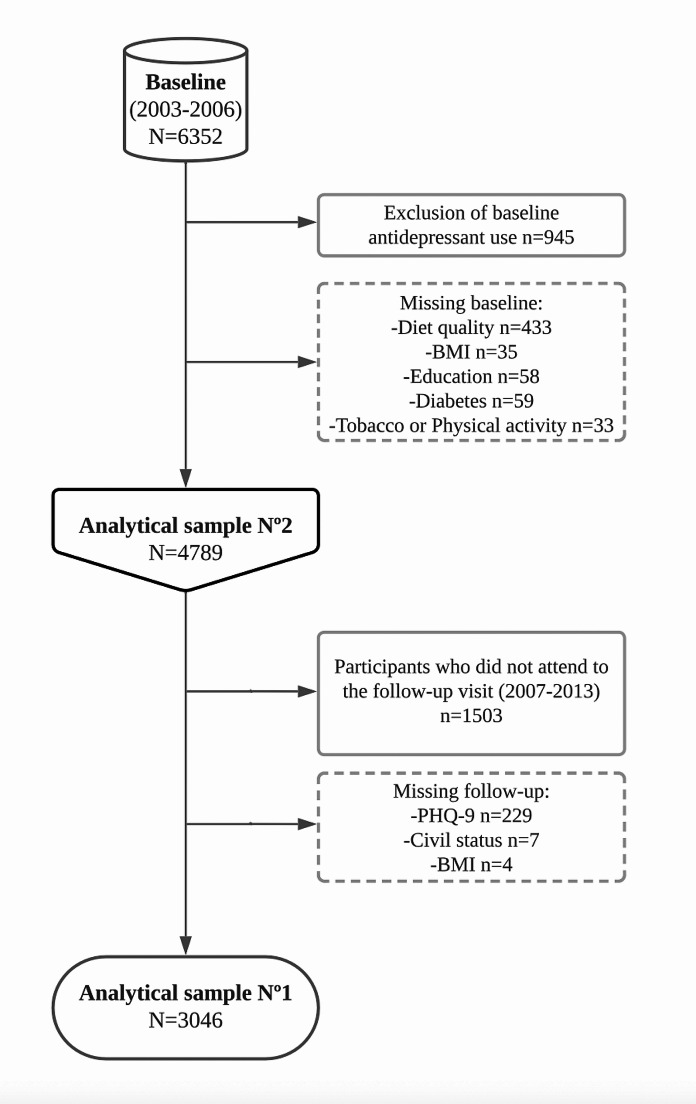
Flow chart of the selection of REGICOR study participants included in the main analysis

For this prospective analysis, we used baseline data from 2003 to 2006 where diet was assessed. To model the incidence of depression over the follow-up, we excluded participants who reported using pharmacological antidepressant treatment at baseline as a proxy for presence of depression. Those participants who had missing data in the food frequency questionnaire (FFQ) or implausible energy intake (< 500 kcal or > 3500 kcal for women and < 800 kcal or > 4000 kcal for men) were excluded. Also, we excluded those who had relevant covariables data missing at baseline. From this initial eligible population two analytical samples were defined (Fig. 1): Analytical sample 1 (*n* = 3,046) included those participants who attended the follow-up visit in 2007–2013 and with available data on the patient health questionnaire (PHQ-9) assessing depression; Analytical sample 2 (*n* = 4,789), included all the initial eligible participants in which a linkage with clinical records was performed (with available clinical diagnosis of depression and date until 31/12/2016). This was used for secondary outcome analysis in order to limit selection bias related to (non-) attendance to the follow-up visit. The STROBE-nutreporting guidelines was used [23].

### Diet assessment

The exposure of interest was measured at baseline using a self-administered 157-item food frequency questionnaire (FFQ) validated in the Spanish population [24]. Food consumption was converted to grams/day and nutritional intake was calculated from nutritional tables, including total energy (kcal), macronutrients (carbohydrates, proteins and lipids), alcohol and micronutrients. Five diet quality scores were computed: the relative Mediterranean Diet (rMED) [25], a Modified Mediterranean Diet Score (ModMDS) [26], the Dietary Approaches to Stop Hypertension (DASH) diet score [27], a Healthful Plant-based Diet Index (HPDI) [28] and the 2015 World Health Organization Healthy Diet Indicator (WHO-HDI) [29]. The scoring criteria and components of each score is described in Table S1. For all of them, a higher score reflects a higher degree of adherence to the dietary guidelines, i.e. better diet quality. These scores were chosen for comparison with the existing literature and for being adapted to our dietary data, as they mostly include food group components and only a limited number of components on nutrient intake, which are less reliably estimated with FFQs.

### Depression assessment

The main outcome was measured at follow-up with the PHQ-9 questionnaire [30], translated and validated in Spanish [31]. This questionnaire is a self-administered version of the Primary Care Evaluation of Mental Disorders diagnostic instrument for common mental disorders. This depression module assesses the presence and frequency in the last 2 weeks of 9 symptom criteria from the Diagnostic and Statistical Manual of Mental Disorders - Fourth Edition (DSM-IV). The 9 questions (Text S1) are scored as follows: 0 = “Not at all”, 1 = “Several days”, 2 = “More than half the days” and 3 = “Nearly every day”, and the final score ranges from 0 to 27 points, in which a higher score indicates more depressive symptoms. To indicate the presence of depressive disorder, a variable was created referring to a person who presents a score ≥ 10, and severe depressive disorder was defined by a score ≥ 15. The PHQ-9 is a well-established validated tool with a sensitivity and specificity of 80–90% for major depression screening [31].

In the Analytical sample 2, a linkage with the electronic health records from the Catalan Government (“PADRIS”) was made, with data from 01/01/2008 to 31/12/2016. We included in the definition of case of depression first occurrence of any of the two following ICD-10 codes [32]: F32 (depressive episode) and F33 (recurrent major depressive disorder). Exit date was date of first diagnosis or censoring date (31/12/2016), whichever came first.

### Covariates

Sociodemographic variables including age, sex, residence area (rural or not), and educational level (low, medium, and high) were collected at baseline and marital status (married/cohabiting, not living with a partner) at the follow-up visit. Participants were asked at baseline about their history of diabetes, hypertension and tobacco use (current, ex-, never smoker). Physical activity was assessed at baseline with the Minnesota leisure-time physical activity questionnaire validated for the Spanish population [33, 34] and expressed in metabolic equivalents of task-minute/day. Clinical information was also collected, including measured body weight and height, blood pressure and serum biomarkers. Body mass index (BMI) was calculated both at baseline and follow-up as weight divided by squared height in meters and expressed as kg·m^− 2^.

All the measurements and the administration of the questionnaires were carried out by trained staff in the participants’ own primary health centers.

### Statistical analysis

Description of baseline characteristics was performed in those included in the main analysis, overall and across quartiles of rMED diet score. Differences across quartiles were assessed by analysis of variance (continuous variables) or chi-square tests (categorical variables). The correlation between the five diet scores was calculated.

In the main analysis, Analytical sample 1 was used. All diet scores were standardized, and sex-specific quartiles were also created. Separate models were fitted for each diet score as explanatory variable (continuous, and in quartiles with the lowest quartile as the reference category). Linear regression models were used to estimate beta coefficients using the PHQ-9 score as a continuous outcome variable. Logistic regression models were used to estimate odds ratios (OR) when the outcome of interest was the presence of depressive disorder (PHQ-9 ≥ 10) at follow-up. For the secondary outcome analysis Analytical sample 2 was used. Multivariable Cox proportional hazards models were used to estimate hazard ratios (HR) with a 95% confidence interval (CI) between diet quality and depression diagnosis. The selection of covariates was based on the literature and the assessment of the association with the exposure and outcome. The same adjustment strategy was used: Model 1 included age, sex, energy intake, residence area, educational level, marital status, diabetes history, tobacco use and physical activity. Model 2 further included baseline BMI and Model 3 further included follow-up BMI. These last two models respond to the objective to test the potential mediating effect of adiposity in the relationship between diet quality and depression, assessed by the attenuation of the coefficients between Model 1 and Models 2 and 3. We provide a descriptive comparison of the baseline characteristics of the participants who were included in the main analyses and those who did not. As sensitivity analysis, we modelled odds of severe depressive disorder (PHQ-9 ≥ 15) in Analytical sample 1.

Two-tailed test P values of less than 0.05 were considered statistically significant. All analyses were performed using R version 4.1.2.

## Results

At baseline, 945 (14.9%) of participants used any antidepressant medication and were excluded. A total of 3,046 adults (50.3% women) with a mean age of 54.7 (standard deviation [SD] = 11.6) years were included in the main analysis (Analytical sample 1, Fig. 1). Compared with participants who were excluded, those included in the main analysis were younger, had lower BMI, were more likely to reside in a rural area, and less likely to be female, to have low education and to have hypertension or diabetes. They did not differ significantly in their tobacco use, and physical activity levels (Table S2).

After a median follow-up of 6.0 years (interquartile range 5.6–6.7 years) between the two visits, 184 (6.04%) participants with incident depression (PHQ-9 ≥ 10) and 50 (1.64%) with severe depression (PHQ-9 ≥ 15) were identified. Characteristics of participants included in the main analysis across quartiles of the rMED are presented in Table 1. Compared to those with lower adherence, participants with higher adherence to rMED (top two quartiles) were older, less likely to smoke, but more likely to have hypertension, diabetes, or a history of cardiovascular disease, and displayed lower energy intake, and higher levels of physical activity. They also displayed lower PHQ-9 scores (fewer depressive symptoms) and lower prevalence of depression. Characteristics across quartiles of the other four dietary scores are presented in Tables S3-S6.

**Table 1:** Baseline characteristics of all REGICOR study participants included in the main analyses by rMED diet score quartiles (*N* = 3046)

	Total	Q1 ^a^	Q2 ^a^	Q3 ^a^	Q4 ^a^	*p*-value ^b^
rMED values: cut-offs	-	[1.00–7.00]	[8.00–9.00]	[10.00–11.00]	[12.00–17.00]	
rMED values: mean (SD) in each quartile	9.2 (2.8)	5.0 (1.1)	7.5 (0.5)	9.5 (0.5)	12.3 (1.3)	< 0.001*
N	3046	506	730	829	981	
Female (%)	1531 (50.3)	271 (53.6)	374 (51.2)	444 (53.6)	442 (45.1)	0.001*
Age (mean (SD))	54.7 (11.6)	49.8 (10.5)	52.7 (11.2)	55.7 (11.6)	58.0 (11.3)	< 0.001*
Educational level (%)						< 0.001*
High	756 (24.8)	117 (23.1)	196 (26.8)	196 (23.6)	247 (25.2)	
Medium	944 (31.0)	185 (36.6)	240 (32.9)	273 (32.9)	246 (25.1)	
Low	1346 (44.2)	204 (40.3)	294 (40.3)	360 (43.4)	488 (49.7)	
Rural residence area (%)	1238 (40.6)	214 (42.3)	304 (41.6)	333 (40.2)	387 (39.4)	0.675
Living with a partner (%)	2360 (77.5)	387 (76.5)	575 (78.8)	639 (77.1)	759 (77.4)	0.786
Smoking status (%)						< 0.001*
Never	1572 (51.6)	255 (50.4)	359 (49.2)	442 (53.3)	516 (52.6)	
Current	660 (21.7)	143 (28.3)	178 (24.4)	166 (20.0)	173 (17.6)	
Ex-smoker	814 (26.7)	108 (21.3)	193 (26.4)	221 (26.7)	292 (29.8)	
Energy kcal/day (mean (SD))	2405.0 (595.7)	2469.6 (615.8)	2421.5 (609.3)	2366.0 (598.6)	2392.2 (569.7)	0.015*
Alcohol consumption gr/day (mean (SD))	13.4 (16.4)	13.3 (19.2)	12.9 (17.4)	12.2 (14.9)	14.7 (15.2)	0.019*
Physical activity METs.min/day (mean (SD))	317.9 (328.3)	266.3 (263.1)	287.9 (311.0)	320.4 (317.7)	364.9 (371.0)	< 0.001*
Baseline Body Mass Index (mean (SD))	27.2 (4.5)	27.1 (4.6)	26.9 (4.6)	27.1 (4.4)	27.4 (4.4)	0.174
Hypertension (%)	1277 (42.1)	165 (32.7)	275 (37.8)	361 (43.9)	476 (48.7)	< 0.001*
Diabetes (%)	325 (10.7)	44 (8.7)	59 (8.1)	85 (10.3)	137 (14.0)	< 0.001*
Previous cardiovascular event (%)	140 (4.6)	11 (2.2)	27 (3.7)	47 (5.7)	55 (5.6)	0.006*
PHQ-9 (mean (SD))	2.8 (3.7)	3.3 (4.1)	2.9 (3.7)	2.7 (3.6)	2.5 (3.4)	0.003*
PHQ-9 ≥ 10 (%)	184 (6.0)	44 (8.7)	43 (5.9)	48 (5.8)	49 (5.0)	0.040*
PHQ-9 ≥ 15 (%)	50 (1.6)	14 (2.8)	16 (2.2)	11 (1.3)	9 (0.9)	0.029*

^a^ Q = quartile of diet quality score

^b^ p-value for the t-test and chi-square test through the quartiles (* significant)

Abbreviations

rMED = Relative Mediterranean Diet

PHQ-9 = Patient Health Questionnaire

**Fig. 2:**
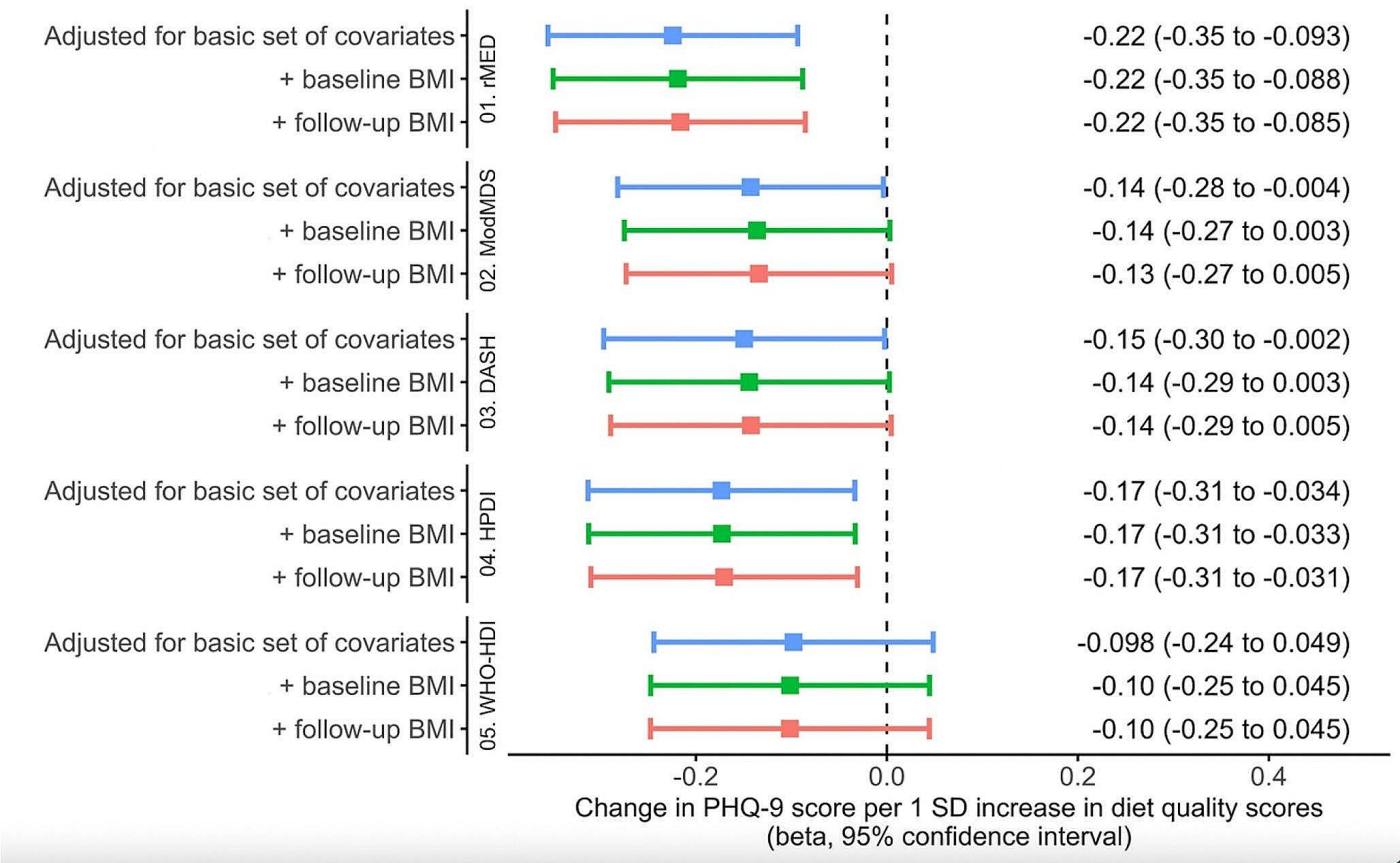
Associations from linear regressions between diet quality scores and PHQ-9 depressive symptoms score disorder in the Analytical sample 1 (*N* = 3046)

**Fig. 3:**
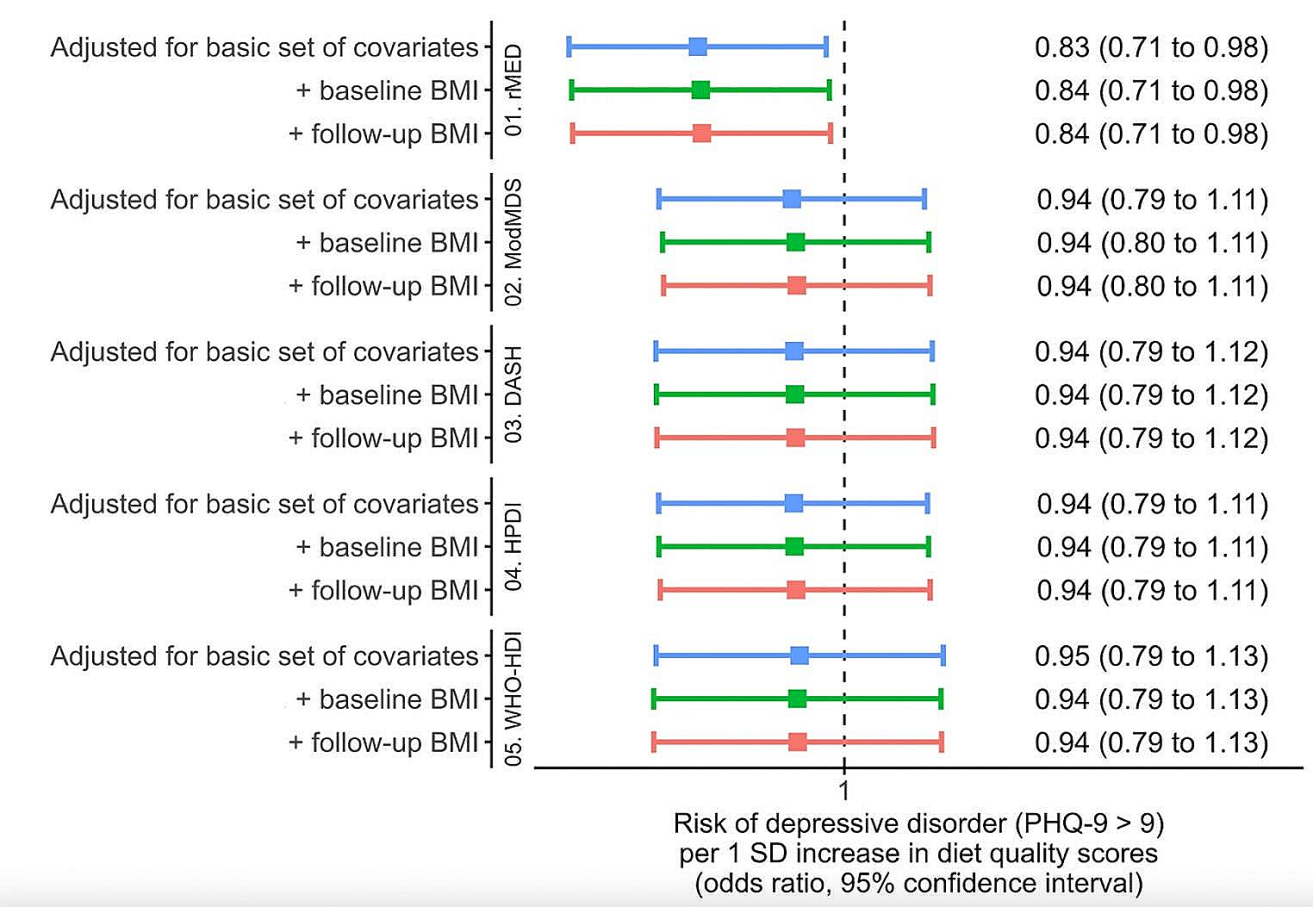
Associations from multivariable logistic regressions between diet quality scores and depressive disorder (PHQ-9 ≥ 10, dichotomous variable) in Analytical sample 1 (*N* = 3046)

**Fig. 4:**
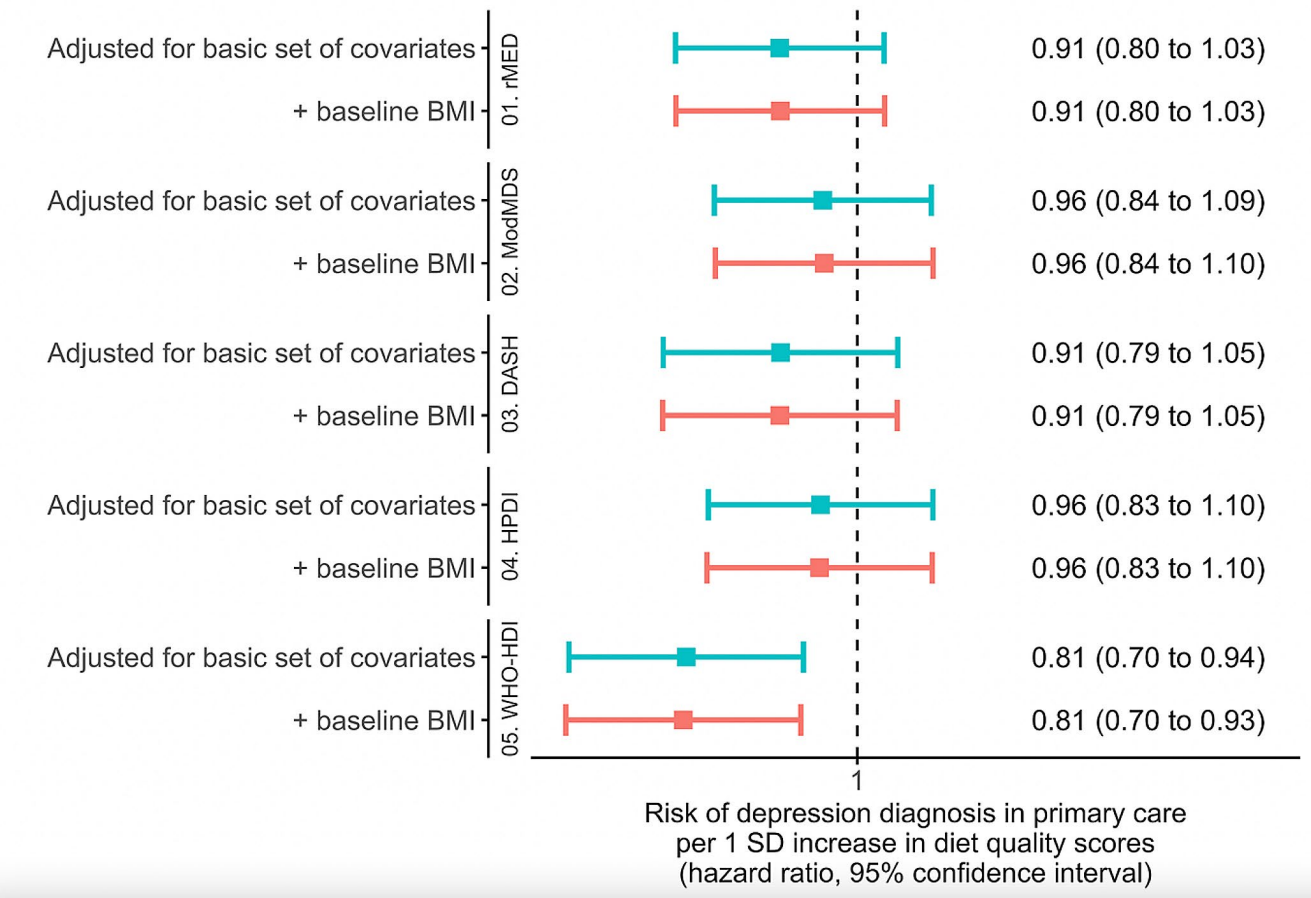
Associations from multivariable Cox proportional hazards models between diet quality scores and depression diagnosis from primary care data in Analytical sample 2 (*N* = 4789)

All diet scores were significantly correlated, ranging from 0.27 (WHO-HDI and HPDI) to 0.69 (ModMDS with both rMED and DASH) (Figure S1). After adjustment for energy intake, age, sex, residence area, educational level, marital status, diabetes history, tobacco use and physical activity (Model 1), all diet scores except the WHO-HDI were associated with fewer depressive symptoms (lower PHQ-9 score), in both continuous and quartile analysis (Fig. 2 and Table S7). rMED had the strongest association with the PHQ-9 score compared to the other diet scores. In quartile analysis, all associations remained unchanged after adjustments for BMI and BMI change at follow-up. In continuous analysis, the ModMDS and the DASH score became non-significant. The relationship with the WHO-HDI score was also inverse but not statistically significant. Only the rMED score was inversely associated with odds of depression (PHQ-9 ≥ 10) (OR per 1 SD increase = 0.84, 95% CI = 0.71–0.98; Fig. 3 and Table S8) and odds of severe depressive disorder (OR per 1 SD increase = 0.70, 95% CI = 0.52–0.95; Figure S2 and Table S9). Adjusting for BMI and BMI change at follow-up did not attenuate the associations. During a median follow-up of 12.15 years (interquartile range 11.13–12.80 years), there were 261 (5.45%) incident cases of depression diagnosis in medical records. The WHO-HDI score was associated with lower risk of depression (HR_model2_=0.81; 95%CI = 0.70–0.93), and weaker, non-significant associations were found for rMED, ModMDS, DASH and HPDI scores (Fig. 4 and Table S10).

## Discussion

In a population-based cohort, rMED, ModMDS, DASH and HPDI diet scores were associated with fewer depressive symptoms at follow-up, and the rMED score was inversely related to the incidence of depression and severe depression (PHQ-9 score ≥ 10 and ≥ 15, respectively). The WHO-HDI score was associated with lower risk of depression based on a clinical diagnosis and inverse association with the other scores were suggested. Adjustment for BMI and BMI change over time did not attenuate the associations.

In recent years, there has been an increase in the literature of longitudinal studies finding a relationship between higher quality diet and lower risk of depression [12], and our results are in line with these findings. However, as seen in our study, different measures of diet quality are not equally associated with depression. For example, in a Dutch prospective study (7-year follow-up), a greater adherence to the Dutch Healthy Diet was associated with a lower risk of depression (HR = 0.83; 95%CI = 0.73–0.96, for every 1 SD increase), however, no association was found with adherence to the Mediterranean diet or the DASH diet [13]. In a Canadian study of adult general population, after a mean follow-up of 14 years, an association was found with both the Healthy Eating Index-Canada and the Modified Mediterranean Diet score but of lower magnitude [20]. These different results between scores could be due to actual different pathophysiological effects of the different dietary components on depression, but also to some scores being better adapted to reflect the actual variability of the diet in the population under study. Other studies did not find significant associations when evaluating their national recommended diet score with the risk of depression, as is the case of the Japan Public Health Center-based prospective study [35] or the Australian Childhood Determinants of Adult Health study [36]. The variability in sociodemographic and health characteristics between populations, the adherence diet scores selected, the way of evaluating the depression outcome, the follow-up time of each cohort are key methodological aspects that may explain the different associations encountered in some studies.

The Mediterranean diet score is the most frequently score evaluated and the one with more consistent inverse associations with depression [12]. In the present study, two different ways of evaluating adherence to the Mediterranean diet were included, as it is the most frequent type of diet in the Spanish population: the rMED [25] and the ModMDS [26]. They include the same main components for their calculation, but in the ModMDS the consumption of each food component is classified into quartiles and in the rMED each component is first calculated based on energy density and then divided into tertiles of consumption. The results of this study using these two scores agree, but we found differences in the magnitude of the association, which was stronger with the rMED. The use of energy density is a way to better grasp the quality of the diet regardless of the quantity consumed and may be a reason why this score shows the strongest statistical association. Overall, our results indicate that the way each Mediterranean diet score is constructed and evaluated is important and influences the results and their interpretation.

Besides differences in their calculation, there are also differences among diet scores regarding some specific components included. The rMED includes a component on olive oil consumption, whereas the ModMDS uses the monounsaturated to saturated fat ratio. This could explain why the results with this score are the most significant, as some observational and clinical trial studies have reported an inverse association between olive oil consumption and the risk of depression [37, 38] and our study population is a population in which olive oil is consumed more frequently as a daily component of the diet. Another differential result was observed with the WHO-HDI score and risk of clinically diagnosed depression. This specific dietary index includes some components not considered in the rest of scores evaluated and this could be an explanation of the observed results. It includes the consumption of free sugar, a detrimental component related to greater inflammation and greater risk of depression [39, 40] and the consumption of dietary fiber, a component associated with lower odds of depression [41, 42]. On the other hand, differences observed between diet scores according to the depressive outcomes used could indicate that in addition to the type of score used to measure the association, the severity of the pathology may also play an important role. Results from the Swedish study [18] also show stronger associations with clinical and severe depression compared to a broader definition of cases.

A key strength of this study is its long-term prospective design. Moreover, it is a population-based cohort of adults residing in the province of Girona - Catalonia, which increases generalizability of the findings to southern Europe adult population, as opposed to some nutritional epidemiology cohorts of heavily selected volunteers. Finally, the measurement of various adherence scores allows the comparison of the results with other similar studies. One of the main limitations of this study is the absence of measures of depressive symptoms or clinical depression at baseline, so that the estimation of the incidence of depression was approximated by excluding those participants under pharmacological antidepressant treatment at the baseline visit as a proxy. It is possible that some participants are undiagnosed or without current pharmacological treatment, or that some people are using antidepressants for other conditions (anxiety, chronic pain, insomnia, or migraine) leading to classification bias, which might result in an underestimation of the association. The main exposure and health outcome of this study were measured using questionnaires (FFQ and PHQ-9), which, despite being validated and widely used, are prone to measurement error and can lead to various types of biases. They are prone to systematic measurement errors that may affect the validity of the data recorded (recall bias and information bias), as well as random errors by which individual characteristics can influence the response to the questionnaire, such as the participant’s body size or mental health state. Selection bias is another limitation to consider, since the presence of depression may be associated with lower participation at the follow-up visit, probably underestimating the incidence, which can bias the existing association with adherence to a healthy diet. However, we were able to somewhat overcome this by using the electronic primary care records for all the participants of the baseline visit, which allowed evaluation of the incidence of depression and the association with the quality of the diet without loss to follow-up. We also cannot rule out residual confounding, and we missed information on potential confounders such as sleep duration or family history of depressive disorders. Finally, as this is an observational study we cannot determine if the association between diet and depression is causal or not. The results of randomized controlled trials carried out in recent years suggest that dietary improvement may provide an efficacious strategy for the prevention and management of depression [43–48]. One large prevention trial (non-depressed participants with overweight) did not find any benefit from the 12-month food-related behavioral activation therapy, although it is important to emphasize that the individuals in that intervention group did not really change their diet throughout the study [49]. More experimental trials with larger representative sample size and longer follow-ups or more than two-time points assessments are needed.

## Conclusions

In this population-based cohort study of Spanish adults, a significant inverse association between diet quality and depression incidence was found. These results would indicate that dietary improvement, as a complementary non-pharmaceutical alternative, may provide an effective, accessible, and cost-effective strategy for the prevention of depression.

Nevertheless, when considering the public health implications of our findings, it is important to remind that depression is a pathology which has structural and systemic causes, and which occurs in many instances regardless of lifestyle factors. Therefore, attributing the disease to a person’s health behaviors would be unfounded and stigmatizing. The responsibility to act and adequately address this pathology falls on political decisions, public health agencies and clinical services together, in order to reduce the high global burden of depression.

## Electronic supplementary material

Below is the link to the electronic supplementary material.

Supplementary Material 1

Supplementary Material 2

Supplementary Material 3

## Data Availability

The datasets analyzed during the current study are not publicly available to protect study participant privacy but could be available from authors upon reasonable request.

## Abbreviations

REGICOR: Registre Gironí del Cor
FFQ: Food Frequency questionnaire
rMED: relative Mediterranean Diet
ModMDS: Modified Mediterranean Diet Score
DASH: Dietary Approaches to Stop Hypertension
HPDI: Healthful Plant-based Indicator
WHO-HDI: 2015 World Health Organization Healthy Diet Indicator
PHQ-9: Patient Health Questionnaire
SD: Standard deviation
OR: Odds Ratio
95%CI: 95% confidence interval
HR: Hazard Ratio
BMI: Body Mass Index
DSM-IV: Diagnostic and Statistical Manual of Mental Disorders, Fourth edition
PADRIS: Programa público de análisis de datos para la investigación e innovación en salud en Catalunya
ICD-10: International Statistical Classification of Diseases, 10th revision
MET: metabolic equivalent of task
