# Supplementary material for: Association between different diet quality scores and depression risk: the REGICOR population-based cohort study
Source: Eur J Nutr. 2024 Aug 24;63(8):2885–95. doi: 10.1007/s00394-024-03466-z (PMC11519306; doi:10.1007/s00394-024-03466-z)
Supplement: Supplementary file 2 — Supplementary Material 2 [file 394_2024_3466_MOESM2_ESM.docx]

**Figure S1. Correlation between diet scores**

**
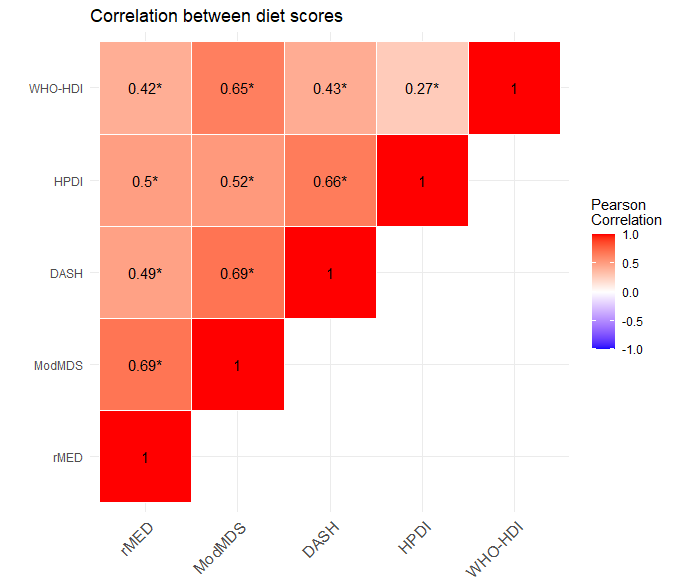
**

* p-value <0.05

**Figure S2.** **Association from multivariable logistic regressions between diet quality scores and severe depressive disorder (PHQ-9 ≥15) in the analytical sample 1 (N=3046)**


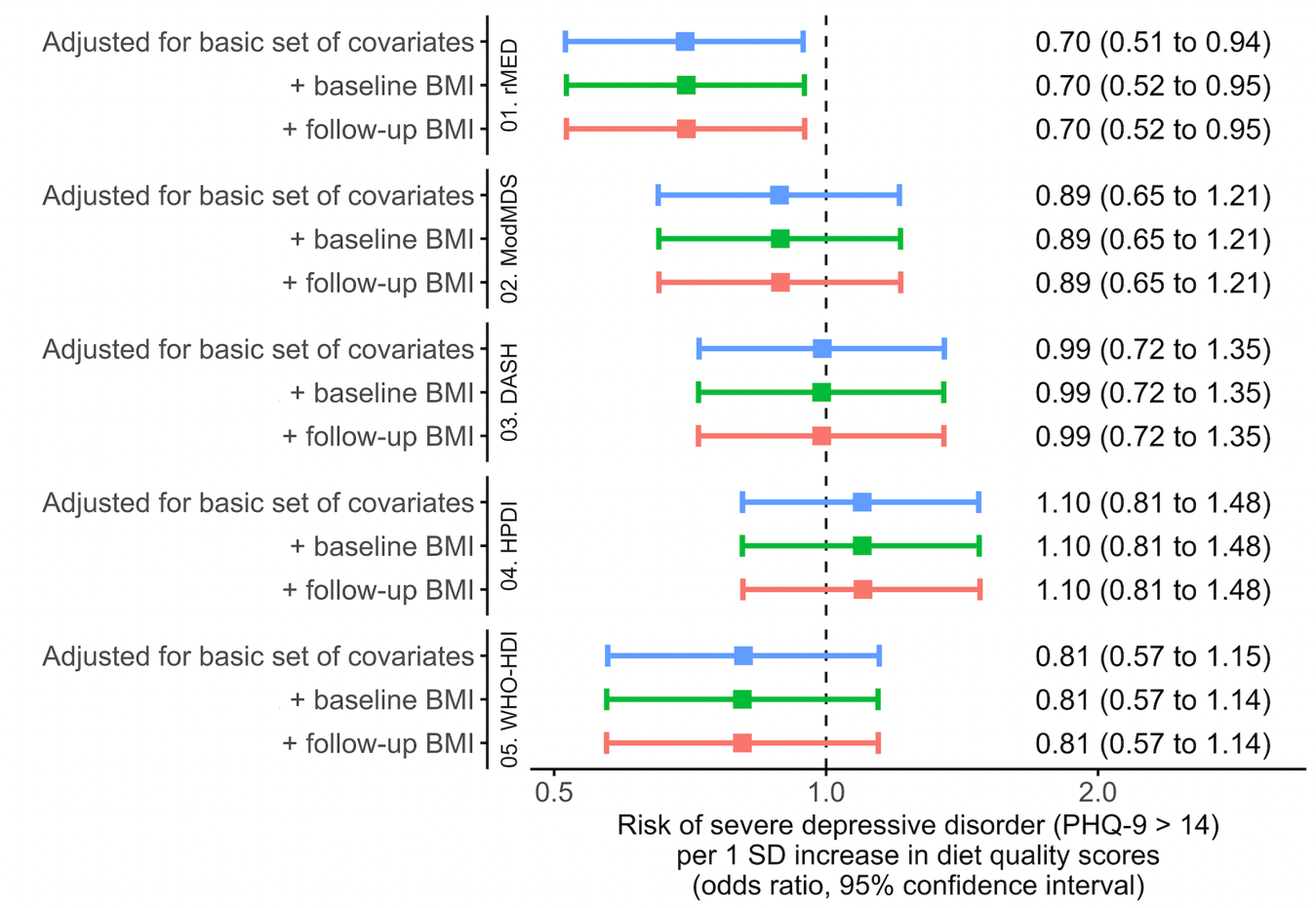


**Table S1. Diet quality score description**

| **Index** | **Scoring** | **Range*** |
| --- | --- | --- |
| Relative Mediterranean Diet (rMED) **(25)** | The consumption of each of the 9 components (apart from alcohol) is calculated as a function of energy density (g · 1000 kcal^-1^ · d^-1^) and then divided into sex-specific tertiles of intakes.   - 5 beneficial (vegetables, legumes, fruits and nuts, cereal, and fish): 0, 1, 2 points are attributed to tertiles 1, 2, 3 - 2 detrimental (meat and poultry, and dairy products): reverse scoring - Fat (intake of olive oil): 0 points for no consumers, 1 for consumption below the median and 2 for equal or above - Alcohol: 2 points if within the consumption range (10-50 g/day for men, 5-25 g/day for women), 0 if outside the range | 0-18 points |
| Modified Mediterranean Diet Score (ModMDS) **(26)** | 9 components classified in sex-specific quartiles of intakes:   - 6 beneficial (vegetables, legumes, fruits, nuts, cereal, and fish) are assigned a score of 0, 1, 2 and 3 from the lowest to the highest quartile - 1 detrimental (red meat): reverse scoring - Fat: MUFA/SFA ratio - Alcohol: 1 point if within the consumption range (10-25 g/day for men, 5-15 g/day for women), 0 if outside the range | 0-25 points |
| The Dietary Approaches to Stop Hypertension (DASH) diet score **(27)** | The consumption of each of the 8 components is classified in sex-specific quintiles of intakes.   - Fruits, vegetables, nuts and legumes, low-fat dairy products, and whole grains: 1 point for quintile 1 and 5 points for quintile 5 - Sodium, red and processed meats, and sweetened beverages: reverse scoring | 8-40 points |
| Healthful Plant-Based Diet Index (HPDI) **(28)** | 18 components classified into 3 food groups. The consumption of each component is classified in sex-specific quintiles   - Healthy Plant Food Groups (whole grains, fruits, vegetables, nuts, legumes, vegetable oils, and tea and coffee): 1 point for quintile 1 and 5 points for quintile 5 - Unhealthy Plant Food Groups (fruit juices, refined grains, potatoes, sugar sweetened beverages, and sweets and desserts): reverse scoring - Animal Food Groups: (animal fat including butter or lard, dairy, eggs, fish and seafood, meat, and miscellaneous animal-based foods): reverse scoring. | 18-90 points |
| 2015 World Health Organization Healthy Diet Indicator (WHO-HDI) **(29)** | 7 components, 1 point is assigned when the intake value is within the recommended range, otherwise 0   - ≥400 g of fruits and vegetables per day - < 30% fat for total energy - < 10% saturated fatty acid for total energy - 6–11% of polyunsaturated fatty acids for total energy - < 10% of free sugar for total energy - ≥25 g/day dietary fiber - ≥3500 mg/ day potassium | 0-7 points |

*Interpretation: Higher scores indicate greater adherence

**Table S2. Comparison of characteristics between participants in the REGICOR study included in the main analyses (Analytical sample 1) and those who were not**

|  | **Included**  **(N=3046)** | **Not included**  **(N=3306)** | **p-value ^a^** |
| --- | --- | --- | --- |
| Female (%) | 1531 (50.3) | 1798 (54.4) | 0.001* |
| Age (mean (SD)) | 54.72 (11.60) | 58.47 (13.18) | <0.001* |
| Educational level (%) |  |  | <0.001* |
| High | 756 (24.8) | 635 (19.7) |  |
| Medium | 944 (31.0) | 813 (25.2) |  |
| Low | 1346 (44.2) | 1779 (55.1) |  |
| Rural residence area (%) | 1238 (40.6) | 1252 (37.9) | 0.025* |
| Smoking status (%) |  |  | 0.660 |
| Never | 1572 (51.6) | 1647 (50.8) |  |
| Current | 660 (21.7) | 733 (22.6) |  |
| Ex-smoker | 814 (26.7) | 864 (26.6) |  |
| Physical activity METs.min/day (mean (SD)) | 317.95 (328.26) | 310.63 (335.66) | 0.380 |
| Baseline BMI (mean (SD)) | 27.16 (4.48) | 27.54 (4.67) | 0.001* |
| Hypertension (%) | 1277 (42.1) | 1568 (50.2) | <0.001* |
| Diabetes (%) | 325 (10.7) | 488 (15.5) | <0.001* |
| Previous cardiovascular event (%) | 140 (4.6) | 259 (7.8) | <0.001* |
| Depression diagnosis from primary care data (%) | 173 (5.7) | 218 (6.6) | 0.144 |

^a^ p-value for the t-test and chi-square test (* significant))

**Table S3. Baseline characteristics of all REGICOR study participants included in the main analyses by ModMDS diet score quartiles (N=3046)**

|  | **Total** | **Q1 ^a^** | **Q2 ^a^** | **Q3 ^a^** | **Q4 ^a^** | **p-value ^b^** |
| --- | --- | --- | --- | --- | --- | --- |
| ModMDS values: cut-offs | - | [0.00 – 10.00] | [11.00 – 13.00] | [14.00 – 16.00] | [17.00 – 25.00] |  |
| ModMDS values: mean (SD) in each quartile | 12.7 (4.3) | 6.8 (1.8) | 10.6 (0.9) | 13.5 (1.0) | 17.6 (1.9) | <0.001* |
| N | 3046 | 657 | 680 | 782 | 927 |  |
| Female (%) | 1531 (50.3) | 327 (49.8) | 322 (47.4) | 395 (50.5) | 487 (52.5) | 0.231 |
| Age (mean (SD)) | 54.7 (11.6) | 50.8 (11.0) | 53.7 (11.4) | 55.4 (11.5) | 57.6 (11.4) | <0.001* |
| Educational level (%) |  |  |  |  |  | <0.001* |
| High | 756 (24.8) | 155 (23.6) | 178 (26.2) | 187 (23.9) | 236 (25.5) |  |
| Medium | 944 (31.0) | 246 (37.4) | 211 (31.0) | 246 (31.5) | 241 (26.0) |  |
| Low | 1346 (44.2) | 256 (39.0) | 291 (42.8) | 349 (44.6) | 450 (48.5) |  |
| Rural residence area (%) | 1238 (40.6) | 274 (41.7) | 286 (42.1) | 320 (40.9) | 358 (38.6) | 0.481 |
| Living with a partner (%) | 2360 (77.5) | 496 (75.5) | 534 (78.5) | 617 (78.9) | 713 (76.9) | 0.394 |
| Smoking status (%) |  |  |  |  |  | <0.001* |
| Never | 1572 (51.6) | 314 (47.8) | 332 (48.8) | 412 (52.7) | 514 (55.4) |  |
| Current | 660 (21.7) | 199 (30.3) | 156 (22.9) | 166 (21.2) | 139 (15.0) |  |
| Ex-smoker | 814 (26.7) | 144 (21.9) | 192 (28.2) | 204 (26.1) | 274 (29.6) |  |
| Energy kcal/day (mean (SD)) | 2405.0 (595.7) | 2179.0 (584.1) | 2299.7 (610.7) | 2450.6 (568.0) | 2603.8 (542.3) | <0.001* |
| Alcohol consumption gr/day (mean (SD)) | 13.4 (16.4) | 14.1 (17.5) | 13.6 (16.3) | 13.9 (18.2) | 12.2 (13.8) | 0.116 |
| Physical activity METs.min/day (mean (SD)) | 317.9 (328.3) | 255.0 (247.7) | 292.1 (333.9) | 332.7 (390.2) | 369.1 (307.5) | <0.001* |
| Baseline Body Mass Index (mean (SD)) | 27.2 (4.5) | 27.0 (4.7) | 27.3 (4.4) | 27.1 (4.6) | 27.2 (4.3) | 0.795 |
| Hypertension (%) | 1277 (42.1) | 227 (34.6) | 287 (42.2) | 340 (43.8) | 423 (46.0) | <0.001* |
| Diabetes (%) | 325 (10.7) | 52 (7.9) | 71 (10.4) | 87 (11.1) | 115 (12.4) | 0.039* |
| Previous cardiovascular event (%) | 140 (4.6) | 24 (3.7) | 27 (4.0) | 38 (4.9) | 51 (5.5) | 0.284 |
| PHQ-9 (mean (SD)) | 2.8 (3.7) | 3.1 (3.8) | 2.7 (3.6) | 2.6 (3.7) | 2.7 (3.6) | 0.045* |
| PHQ-9 ≥ 10 (%) | 184 (6.0) | 50 (7.6) | 38 (5.6) | 41 (5.2) | 55 (5.9) | 0.262 |
| PHQ-9 ≥ 15 (%) | 50 (1.6) | 12 (1.8) | 12 (1.8) | 16 (2.0) | 10 (1.1) | 0.421 |

^a^ Q = quartile of diet quality score

^b^ p-value for the t-test and chi-square test through the quartiles (* significant)

Abbreviations

ModMDS = Modified Mediterranean Diet Score

PHQ-9 = Patient Health Questionnaire

**Table S4. Baseline characteristics of all REGICOR study participants included in the main analyses by DASH diet score quartiles (N=3046)**

|  | **Total** | **Q1 ^a^** | **Q2 ^a^** | **Q3 ^a^** | **Q4 ^a^** | **p-value ^b^** |
| --- | --- | --- | --- | --- | --- | --- |
| DASH values: cut-offs | - | [8.00 – 20.00] | [21.00 – 24.00] | [25.00 – 27.00] | [28.00 – 38.00] |  |
| DASH values: mean (SD) in each quartile | 23.8 (5.2) | 17.5 (3.1) | 21.7 (2.5) | 25.0 (2.1) | 29.5 (2.9) | <0.001* |
| N | 3046 | 664 | 791 | 698 | 893 |  |
| Female (%) | 1531 (50.3) | 298 (44.9) | 430 (54.4) | 343 (49.1) | 460 (51.5) | 0.003* |
| Age (mean (SD)) | 54.7 (11.6) | 48.6 (9.6) | 52.7 (10.9) | 56.7 (11.6) | 59.6 (11.1) | <0.001* |
| Educational level (%) |  |  |  |  |  | <0.001* |
| High | 756 (24.8) | 170 (25.6) | 209 (26.4) | 167 (23.9) | 210 (23.5) |  |
| Medium | 944 (31.0) | 253 (38.1) | 260 (32.9) | 210 (30.1) | 221 (24.7) |  |
| Low | 1346 (44.2) | 241 (36.3) | 322 (40.7) | 321 (46.0) | 462 (51.7) |  |
| Rural residence area (%) | 1238 (40.6) | 288 (43.4) | 356 (45.0) | 266 (38.1) | 328 (36.7) | 0.001* |
| Living with a partner (%) | 2360 (77.5) | 517 (77.9) | 640 (80.9) | 531 (76.1) | 672 (75.3) | 0.033* |
| Smoking status (%) |  |  |  |  |  | <0.001* |
| Never | 1572 (51.6) | 282 (42.5) | 425 (53.7) | 361 (51.7) | 504 (56.4) |  |
| Current | 660 (21.7) | 217 (32.7) | 178 (22.5) | 136 (19.5) | 129 (14.4) |  |
| Ex-smoker | 814 (26.7) | 165 (24.8) | 188 (23.8) | 201 (28.8) | 260 (29.1) |  |
| Energy kcal/day (mean (SD)) | 2405.0 (595.7) | 2501.4 (588.7) | 2368.8 (604.8) | 2376.5 (620.7) | 2387.6 (565.7) | <0.001* |
| Alcohol consumption gr/day (mean (SD)) | 13.4 (16.4) | 15.8 (18.5) | 14.1 (16.9) | 12.9 (16.4) | 11.1 (13.6) | <0.001* |
| Physical activity METs.min/day (mean (SD)) | 317.9 (328.3) | 276.3 (323.6) | 280.7 (270.6) | 335.8 (390.9) | 367.9 (317.0) | <0.001* |
| Baseline Body Mass Index (mean (SD)) | 27.2 (4.5) | 26.9 (4.5) | 27.0 (4.6) | 27.3 (4.3) | 27.5 (4.4) | 0.036* |
| Hypertension (%) | 1277 (42.1) | 197 (29.7) | 316 (40.1) | 319 (46.0) | 445 (50.2) | <0.001* |
| Diabetes (%) | 325 (10.7) | 34 (5.1) | 75 (9.5) | 85 (12.2) | 131 (14.7) | <0.001* |
| Previous cardiovascular event (%) | 140 (4.6) | 11 (1.7) | 26 (3.3) | 34 (4.9) | 69 (7.7) | <0.001* |
| PHQ-9 (mean (SD)) | 2.8 (3.7) | 3.0 (3.6) | 2.8 (3.8) | 2.6 (3.6) | 2.7 (3.6) | 0.342 |
| PHQ-9 ≥ 10 (%) | 184 (6.0) | 41 (6.2) | 53 (6.7) | 37 (5.3) | 53 (5.9) | 0.725 |
| PHQ-9 ≥ 15 (%) | 50 (1.6) | 9 (1.4) | 16 (2.0) | 11 (1.6) | 14 (1.6) | 0.778 |

^a^ Q = quartile of diet quality score

^b^ p-value for the t-test and chi-square test through the quartiles (* significant)

Abbreviations

DASH = Dietary Approaches to Stop Hypertension

PHQ-9 = Patient Health Questionnaire

**Table S5. Baseline characteristics of all REGICOR study participants included in the main analyses by HPDI diet score quartiles (N=3046)**

|  | **Total** | **Q1 ^a^** | **Q2 ^a^** | **Q3 ^a^** | **Q4 ^a^** | **p-value ^b^** |
| --- | --- | --- | --- | --- | --- | --- |
| HPDI values: cut-offs | - | [30.00 – 47.00] | [48.00 – 52.00] | [53.00 – 57.00] | [58.00 – 79.00] |  |
| HPDI values: mean (SD) in each quartile | 52.2 (6.8) | 42.9 (3.0) | 49.2 (1.7) | 53.8 (1.8) | 60.4 (3.4) | <0.001* |
| N | 3046 | 635 | 770 | 810 | 831 |  |
| Female (%) | 1531 (50.3) | 324 (51.0) | 382 (49.6) | 420 (51.9) | 405 (48.7) | 0.600 |
| Age (mean (SD)) | 54.7 (11.6) | 49.0 (10.5) | 52.9 (11.2) | 56.3 (11.4) | 59.3 (10.8) | <0.001* |
| Educational level (%) |  |  |  |  |  | <0.001* |
| High | 756 (24.8) | 187 (29.4) | 178 (23.1) | 195 (24.1) | 196 (23.6) |  |
| Medium | 944 (31.0) | 228 (35.9) | 257 (33.4) | 238 (29.4) | 221 (26.6) |  |
| Low | 1346 (44.2) | 220 (34.6) | 335 (43.5) | 377 (46.5) | 414 (49.8) |  |
| Rural residence area (%) | 1238 (40.6) | 283 (44.6) | 334 (43.4) | 317 (39.1) | 304 (36.6) | 0.005* |
| Living with a partner (%) | 2360 (77.5) | 486 (76.5) | 617 (80.1) | 627 (77.4) | 630 (75.8) | 0.191 |
| Smoking status (%) |  |  |  |  |  | <0.001* |
| Never | 1572 (51.6) | 320 (50.4) | 407 (52.9) | 431 (53.2) | 414 (49.8) |  |
| Current | 660 (21.7) | 166 (26.1) | 183 (23.8) | 168 (20.7) | 143 (17.2) |  |
| Ex-smoker | 814 (26.7) | 149 (23.5) | 180 (23.4) | 211 (26.0) | 274 (33.0) |  |
| Energy kcal/day (mean (SD)) | 2405.0 (595.7) | 2645.7 (533.6) | 2496.7 (573.1) | 2338.4 (585.1) | 2200.9 (590.3) | <0.001* |
| Alcohol consumption gr/day (mean (SD)) | 13.4 (16.4) | 13.5 (16.4) | 14.3 (17.5) | 13.1 (17.0) | 12.7 (14.7) | 0.287 |
| Physical activity METs.min/day (mean (SD)) | 317.9 (328.3) | 269.1 (318.2) | 302.9 (315.0) | 321.9 (259.3) | 365.4 (395.3) | <0.001* |
| Baseline Body Mass Index (mean (SD)) | 27.2 (4.5) | 26.6 (4.5) | 27.0 (4.7) | 27.5 (4.3) | 27.4 (4.4) | <0.001* |
| Hypertension (%) | 1277 (42.1) | 198 (31.2) | 309 (40.2) | 359 (44.5) | 411 (49.9) | <0.001* |
| Diabetes (%) | 325 (10.7) | 33 (5.2) | 64 (8.3) | 103 (12.7) | 125 (15.0) | <0.001* |
| Previous cardiovascular event (%) | 140 (4.6) | 9 (1.4) | 30 (3.9) | 37 (4.6) | 64 (7.7) | <0.001* |
| PHQ-9 (mean (SD)) | 2.8 (3.7) | 3.0 (3.7) | 2.9 (3.7) | 2.7 (3.6) | 2.7 (3.6) | 0.160 |
| PHQ-9 ≥ 10 (%) | 184 (6.0) | 40 (6.3) | 50 (6.5) | 43 (5.3) | 51 (6.1) | 0.770 |
| PHQ-9 ≥ 15 (%) | 50 (1.6) | 13 (2.0) | 10 (1.3) | 15 (1.9) | 12 (1.4) | 0.652 |

^a^ Q = quartile of diet quality score

^b^ p-value for the t-test and chi-square test through the quartiles (* significant)

Abbreviations

HPDI = Healthful Plant-based Diet Index

PHQ-9 = Patient Health Questionnaire

**Table S6. Baseline characteristics of all REGICOR study participants included in the main analyses by WHO-HDI diet score quartiles (N=3046)**

|  | **Total** | **Q1 ^a^** | **Q2 ^a^** | **Q3 ^a^** | **Q4 ^a^** | **p-value ^b^** |
| --- | --- | --- | --- | --- | --- | --- |
| WHO-HDI values: cut-offs | - | [0.00 – 2.00] | [3.00] | [4.00] | [5.00 - 6.00] |  |
| WHO-HDI values: mean (SD) in each quartile | 2.8 (1.3) | 0.8 (0.4) | 2.0 (0.0) | 3.0 (0.0) | 4.2 (0.4) | <0.001* |
| N | 3046 | 508 | 613 | 964 | 961 |  |
| Female (%) | 1531 (50.3) | 196 (38.6) | 312 (50.9) | 526 (54.6) | 497 (51.7) | <0.001* |
| Age (mean (SD)) | 54.7 (11.6) | 52.2 (11.6) | 53.5 (11.4) | 54.5 (11.7) | 57.1 (11.2) | <0.001* |
| Educational level (%) |  |  |  |  |  | <0.001* |
| High | 756 (24.8) | 141 (27.8) | 151 (24.6) | 255 (26.5) | 209 (21.7) |  |
| Medium | 944 (31.0) | 181 (35.6) | 213 (34.7) | 306 (31.7) | 244 (25.4) |  |
| Low | 1346 (44.2) | 186 (36.6) | 249 (40.6) | 403 (41.8) | 508 (52.9) |  |
| Rural residence area (%) | 1238 (40.6) | 215 (42.3) | 261 (42.6) | 381 (39.5) | 381 (39.6) | 0.486 |
| Living with a partner (%) | 2360 (77.5) | 391 (77.0) | 468 (76.3) | 746 (77.4) | 755 (78.6) | 0.758 |
| Smoking status (%) |  |  |  |  |  | <0.001* |
| Never | 1572 (51.6) | 220 (43.3) | 302 (49.3) | 515 (53.4) | 535 (55.7) |  |
| Current | 660 (21.7) | 152 (29.9) | 154 (25.1) | 202 (21.0) | 152 (15.8) |  |
| Ex-smoker | 814 (26.7) | 136 (26.8) | 157 (25.6) | 247 (25.6) | 274 (28.5) |  |
| Energy kcal/day (mean (SD)) | 2405.0 (595.7) | 1952.4 (506.9) | 2193.1 (530.4) | 2538.7 (547.5) | 2645.2 (545.3) | <0.001* |
| Alcohol consumption gr/day (mean (SD)) | 13.4 (16.4) | 14.6 (16.0) | 14.2 (17.0) | 13.7 (17.9) | 11.8 (14.4) | 0.009* |
| Physical activity METs.min/day (mean (SD)) | 317.9 (328.3) | 285.0 (402.2) | 269.7 (251.6) | 330.3 (346.3) | 353.7 (303.7) | <0.001* |
| Baseline Body Mass Index (mean (SD)) | 27.2 (4.5) | 26.9 (4.2) | 27.1 (4.5) | 27.1 (4.4) | 27.4 (4.7) | 0.210 |
| Hypertension (%) | 1277 (42.1) | 181 (35.6) | 256 (41.9) | 386 (40.3) | 454 (47.5) | <0.001* |
| Diabetes (%) | 325 (10.7) | 43 (8.5) | 54 (8.8) | 99 (10.3) | 129 (13.4) | 0.006* |
| Previous cardiovascular event (%) | 140 (4.6) | 19 (3.7) | 27 (4.4) | 33 (3.4) | 61 (6.3) | 0.014* |
| PHQ-9 (mean (SD)) | 2.8 (3.7) | 2.8 (3.8) | 2.7 (3.4) | 2.8 (3.7) | 2.8 (3.6) | 0.895 |
| PHQ-9 ≥ 10 (%) | 184 (6.0) | 30 (5.9) | 34 (5.5) | 62 (6.4) | 58 (6.0) | 0.910 |
| PHQ-9 ≥ 15 (%) | 50 (1.6) | 12 (2.4) | 4 (0.7) | 19 (2.0) | 15 (1.6) | 0.110 |

^a^ Q = quartile of diet quality score

^b^ p-value for the t-test and chi-square test through the quartiles (* significant)

Abbreviations

WHO-HDI = World Health Organization Healthy Diet Indicator

PHQ-9 = Patient Health Questionnaire

**Table S7. Associations from linear regressions between diet quality scores (continuous and quartiles) and PHQ-9 depression score disorder in the Analytical sample 1 (N=3046)**

| **rMED** | Continuous^a,b^ | Q1^a,c^ | Q2^a,c^ | Q3^a,c^ | Q4^a,c^ | P_trend_ |
| --- | --- | --- | --- | --- | --- | --- |
| Model 1^d^ | -0.22 (-0.35; -0.09) | (Reference) | -0.32 (-0.72; 0.08) | -0.49 (-0.89; -0.10) | -0.60 (-1.00; -0.21) | 0.002* |
| Model 2^e^ | -0.22 (-0.35; -0.09) | (Reference) | -0.31 (-0.71; 0.09) | -0.48 (-0.88; -0.08) | -0.59 (-0.98; -0.20) | 0.003* |
| Model 3^f^ | -0.22 (-0.35; -0.09) | (Reference) | -0.31 (-0.71; 0.09) | -0.48 (-0.87; -0.08) | -0.58 (-0.98; -0.19) | 0.003* |
|  | | | | | |  |
| **ModMDS** | Continuous^a,b^ | Q1^a,c^ | Q2^a,c^ | Q3^a,c^ | Q4^a,c^ |  |
| Model 1^d^ | -0.14 (-0.28; -0.00) | (Reference) | -0.40 (-0.79; -0.02) | -0.54 (-0.92; -0.16) | -0.54 (-0.93; -0.16) | 0.007* |
| Model 2^e^ | -0.14 (-0.27; 0.00) | (Reference) | -0.40 (-0.79; -0.02) | -0.53 (-0.91; -0.15) | -0.53 (-0.92; -0.15) | 0.009* |
| Model 3^f^ | -0.13 (-0.27; 0.01) | (Reference) | -0.41 (-0.79; -0.03) | -0.53 (-0.91; -0.15) | -0.53 (-0.91; -0.14) | 0.010* |
|  | | | | | |  |
| **DASH** | Continuous^a,b^ | Q1^a,c^ | Q2^a,c^ | Q3^a,c^ | Q4^a,c^ |  |
| Model 1^d^ | -0.15 (-0.30; -0.00) | (Reference) | -0.32 (-0.69; 0.05) | -0.44 (-0.83; -0.05) | -0.41 (-0.79; -0.03) | 0.041* |
| Model 2^e^ | -0.14 (-0.29; 0.00) | (Reference) | -0.32 (-0.69; 0.05) | -0.43 (-0.82; -0.04) | -0.40 (-0.78; -0.02) | 0.046* |
| Model 3^f^ | -0.14 (-0.29; 0.00) | (Reference) | -0.31 (-0.68; 0.06) | -0.43 (-0.82; -0.04) | -0.40 (-0.78; -0.02) | 0.048* |
|  | | | | | |  |
| **HPDI** | Continuous^a,b^ | Q1^a,c^ | Q2^a,c^ | Q3^a,c^ | Q4^a,c^ |  |
| Model 1^d^ | -0.17 (-0.31; -0.03) | (Reference) | -0.16 (-0.53; 0.22) | -0.46 (-0.84; -0.07) | -0.41 (-0.81; -0.02) | 0.018* |
| Model 2^e^ | -0.17 (-0.31; -0.03) | (Reference) | -0.16 (-0.54; 0.21) | -0.47 (-0.85; -0.09) | -0.41 (-0.81; -0.02) | 0.018* |
| Model 3^f^ | -0.17 (-0.31; -0.03) | (Reference) | -0.16 (-0.53; 0.22) | -0.46 (-0.85; -0.08) | -0.41 (-0.80; -0.01) | 0.019* |
|  | | | | | |  |
| **WHO-HDI** | Continuous^a,b^ | Q1^a,c^ | Q2^a,c^ | Q3^a,c^ | Q4^a,c^ |  |
| Model 1^d^ | -0.10 (-0.24; 0.05) | (Reference) | -0.36 (-0.78; 0.06) | -0.31 (-0.72; 0.11) | -0.35 (-0.78; 0.09) | 0.223 |
| Model 2^e^ | -0.10 (-0.25; 0.04) | (Reference) | -0.37 (-0.80; 0.05) | -0.33 (-0.74; 0.09) | -0.37 (-0.80; 0.07) | 0.196 |
| Model 3^f^ | -0.10 (-0.25; 0.04) | (Reference) | -0.38 (-0.80; 0.04) | -0.34 (-0.76; 0.08) | -0.37 (-0.81; 0.07) | 0.190 |

^a^ Values are beta coefficients (95% confidence interval; p-value (* significant))

^b^ Beta coefficient for the increase in 1 standard deviation of diet quality score

^c^ Q= quartile of diet quality score

^d^ Model 1 includes age, sex, energy intake, residence area, educational level, marital status, diabetes history, tobacco use and physical activity

^e^ Model 2 further includes baseline BMI

^f^ Model 3 further includes follow-up BMI

Abbreviations

rMED = Relative Mediterranean Diet

ModMDS = Modified Mediterranean Diet Score

DASH = Dietary Approaches to Stop Hypertension

HPDI = Healthful Plant-based Diet Index

WHO-HDI = World Health Organization Healthy Diet Indicator

Ptrend = p-value for linear trend across quartiles

**Table S8. Associations from multivariable logistic regressions between diet quality scores (continuous and quartiles) and depressive disorder (PHQ-9 ≥10, dichotomous variable) in Analytical sample 1 (N=3046)**

| **rMED** | Continuous^a,b^ | Q1^a,c^ | Q2^a,c^ | Q3^a,c^ | Q4^a,c^ | P_trend_ |
| --- | --- | --- | --- | --- | --- | --- |
| N cases / N total | - | 44 / 506 | 43 / 730 | 48 / 829 | 49 / 981 |  |
| Model 1^d^ | 0.83 (0.71; 0.98) | 1.00 (Reference) | 0.70 (0.45; 1.10) | 0.68 (0.43; 1.05) | 0.61 (0.39; 0.95) | 0.042* |
| Model 2^e^ | 0.84 (0.71; 0.98) | 1.00 (Reference) | 0.70 (0.45; 1.10) | 0.68 (0.44; 1.06) | 0.61 (0.39; 0.96) | 0.047* |
| Model 3^f^ | 0.84 (0.71; 0.98) | 1.00 (Reference) | 0.70 (0.45; 1.10) | 0.68 (0.44; 1.07) | 0.61 (0.39; 0.96) | 0.050 |
|  | | | | | |  |
| **ModMDS** | Continuous^a,b^ | Q1^a,c^ | Q2^a,c^ | Q3^a,c^ | Q4^a,c^ |  |
| N cases / N total | - | 50 / 657 | 38 / 680 | 41 / 782 | 55 / 927 |  |
| Model 1^d^ | 0.94 (0.79; 1.11) | 1.00 (Reference) | 0.74 (0.47; 1.16) | 0.69 (0.44; 1.08) | 0.75 (0.48; 1.18) | 0.229 |
| Model 2^e^ | 0.94 (0.80; 1.11) | 1.00 (Reference) | 0.74 (0.47; 1.17) | 0.69 (0.44; 1.08) | 0.76 (0.49; 1.19) | 0.252 |
| Model 3^f^ | 0.94 (0.80; 1.11) | 1.00 (Reference) | 0.74 (0.47; 1.16) | 0.69 (0.44; 1.08) | 0.77 (0.49; 1.20) | 0.260 |
|  | | | | | |  |
| **DASH** | Continuous^a,b^ | Q1^a,c^ | Q2^a,c^ | Q3^a,c^ | Q4^a,c^ |  |
| N cases / N total | - | 41 / 664 | 53 / 791 | 37 / 698 | 53 / 893 |  |
| Model 1^d^ | 0.94 (0.79; 1.12) | 1.00 (Reference) | 1.01 (0.65; 1.56) | 0.80 (0.49; 1.29) | 0.88 (0.56; 1.39) | 0.430 |
| Model 2^e^ | 0.94 (0.79; 1.12) | 1.00 (Reference) | 1.01 (0.65; 1.57) | 0.80 (0.49; 1.29) | 0.88 (0.56; 1.39) | 0.427 |
| Model 3^f^ | 0.94 (0.79; 1.12) | 1.00 (Reference) | 1.01 (0.66; 1.57) | 0.80 (0.49; 1.29) | 0.88 (0.56; 1.40) | 0.428 |
|  | | | | | |  |
| **HPDI** | Continuous^a,b^ | Q1^a,c^ | Q2^a,c^ | Q3^a,c^ | Q4^a,c^ |  |
| N cases / N total | - | 40 / 635 | 50 / 770 | 43 / 810 | 51 / 831 |  |
| Model 1^d^ | 0.94 (0.79; 1.11) | 1.00 (Reference) | 1.03 (0.66; 1.62) | 0.81 (0.51; 1.29) | 0.97 (0.61; 1.56) | 0.668 |
| Model 2^e^ | 0.94 (0.79; 1.11) | 1.00 (Reference) | 1.03 (0.66; 1.61) | 0.80 (0.50; 1.27) | 0.97 (0.61; 1.56) | 0.661 |
| Model 3^f^ | 0.94 (0.79; 1.11) | 1.00 (Reference) | 1.03 (0.66; 1.62) | 0.80 (0.50; 1.27) | 0.97 (0.61; 1.56) | 0.671 |
|  | | | | | |  |
| **WHO-HDI** | Continuous^a,b^ | Q1^a,c^ | Q2^a,c^ | Q3^a,c^ | Q4^a,c^ |  |
| N cases / N total | - | 30 / 508 | 34 / 613 | 62 / 964 | 58 / 961 |  |
| Model 1^d^ | 0.95 (0.79; 1.13) | 1.00 (Reference) | 0.80 (0.47; 1.35) | 0.95 (0.58; 1.59) | 0.87 (0.52; 1.49) | 0.817 |
| Model 2^e^ | 0.94 (0.79; 1.13) | 1.00 (Reference) | 0.79 (0.47; 1.34) | 0.94 (0.57; 1.57) | 0.86 (0.51; 1.47) | 0.780 |
| Model 3^f^ | 0.94 (0.79; 1.13) | 1.00 (Reference) | 0.79 (0.47; 1.33) | 0.93 (0.57; 1.56) | 0.86 (0.51; 1.47) | 0.778 |

^a^ Values are odds ratios (95% confidence interval; p-value (* significant))

^b^ Odds ratio for the increase in 1 standard deviation of diet quality score

^c^ Q= quartile of diet quality score

^d^ Model 1 includes age, sex, energy intake, residence area, educational level, marital status, diabetes history, tobacco use and physical activity

^e^ Model 2 further includes baseline BMI

^f^ Model 3 further includes follow-up BMI

Abbreviations

rMED = Relative Mediterranean Diet

ModMDS = Modified Mediterranean Diet Score

DASH = Dietary Approaches to Stop Hypertension

HPDI = Healthful Plant-based Diet Index

WHO-HDI = World Health Organization Healthy Diet Indicator

Ptrend = p-value for linear trend across quartiles

**Table S9.** **Association from multivariable logistic regressions between diet quality scores (continuous and quartiles) and severe depressive disorder (PHQ-9 ≥15) in the analytical sample 1 (N=3046)**

| **rMED** | Continuous^a,b^ | Q1^a,c^ | Q2^a,c^ | Q3^a,c^ | Q4^a,c^ | P_trend_ |
| --- | --- | --- | --- | --- | --- | --- |
| N cases / N total | - | 14 / 506 | 16 / 730 | 11 / 829 | 9 / 981 |  |
| Model 1^d^ | 0.70 (0.51;0.94) | 1.00 (Reference) | 0.95 (0.45;2.02) | 0.60 (0.26;1.37) | 0.47 (0.19;1.12) | 0.053 |
| Model 2^e^ | 0.70 (0.52;0.95) | 1.00 (Reference) | 0.95 (0.45;2.02) | 0.60 (0.26;1.38) | 0.47 (0.19;1.13) | 0.055 |
| Model 3^f^ | 0.70 (0.52;0.95) | 1.00 (Reference) | 0.94 (0.45;2.02) | 0.60 (0.26;1.38) | 0.48 (0.19;1.13) | 0.056 |
|  | | | | | |  |
| **ModMDS** | Continuous^a,b^ | Q1^a,c^ | Q2^a,c^ | Q3^a,c^ | Q4^a,c^ |  |
| N cases / N total | - | 12 / 657 | 12 / 680 | 16 / 782 | 10 / 927 |  |
| Model 1^d^ | 0.89 (0.65;1.21) | 1.00 (Reference) | 1.14 (0.49;2.66) | 1.36 (0.61;3.10) | 0.67 (0.26;1.69) | 0.503 |
| Model 2^e^ | 0.89 (0.65;1.21) | 1.00 (Reference) | 1.15 (0.49;2.68) | 1.36 (0.61;3.11) | 0.68 (0.27;1.71) | 0.518 |
| Model 3^f^ | 0.89 (0.65;1.21) | 1.00 (Reference) | 1.15 (0.49;2.67) | 1.36 (0.61;3.10) | 0.68 (0.27;1.71) | 0.523 |
|  | | | | | |  |
| **DASH** | Continuous^a,b^ | Q1^a,c^ | Q2^a,c^ | Q3^a,c^ | Q4^a,c^ |  |
| N cases / N total | - | 9 / 664 | 16 / 791 | 11 / 698 | 14 / 893 |  |
| Model 1^d^ | 0.99 (0.72;1.35) | 1.00 (Reference) | 1.56 (0.68;3.80) | 1.36 (0.54;3.52) | 1.41 (0.58;3.59) | 0.607 |
| Model 2^e^ | 0.99 (0.72;1.35) | 1.00 (Reference) | 1.56 (0.68;3.79) | 1.36 (0.54;3.50) | 1.40 (0.57;3.56) | 0.621 |
| Model 3^f^ | 0.99 (0.72;1.35) | 1.00 (Reference) | 1.56 (0.68;3.80) | 1.35 (0.54;3.50) | 1.40 (0.58;3.56) | 0.621 |
|  | | | | | |  |
| **HPDI** | Continuous^a,b^ | Q1^a,c^ | Q2^a,c^ | Q3^a,c^ | Q4^a,c^ |  |
| N cases / N total | - | 13 / 635 | 10 / 770 | 15 / 810 | 12 / 831 |  |
| Model 1^d^ | 1.10 (0.81;1.48) | 1.00 (Reference) | 0.72 (0.30;1.69) | 1.17 (0.53;2.62) | 1.07 (0.45;2.55) | 0.619 |
| Model 2^e^ | 1.10 (0.81;1.48) | 1.00 (Reference) | 0.72 (0.30;1.68) | 1.15 (0.52;2.59) | 1.07 (0.45;2.54) | 0.628 |
| Model 3^f^ | 1.10 (0.81;1.48) | 1.00 (Reference) | 0.72 (0.30;1.69) | 1.15 (0.52;2.59) | 1.07 (0.45;2.55) | 0.624 |
|  | | | | | |  |
| **WHO-HDI** | Continuous^a,b^ | Q1^a,c^ | Q2^a,c^ | Q3^a,c^ | Q4^a,c^ |  |
| N cases / N total | - | 12 / 508 | 4 / 613 | 19 / 964 | 15 / 961 |  |
| Model 1^d^ | 0.81 (0.57;1.15) | 1.00 (Reference) | 0.20 (0.05;0.59) | 0.63 (0.27;1.51) | 0.52 (0.20;1.32) | 0.537 |
| Model 2^e^ | 0.81 (0.57;1.14) | 1.00 (Reference) | 0.19 (0.05;0.58) | 0.62 (0.27;1.49) | 0.51 (0.20;1.31) | 0.523 |
| Model 3^f^ | 0.81 (0.57;1.14) | 1.00 (Reference) | 0.19 (0.05;0.58) | 0.62 (0.26;1.48) | 0.51 (0.20;1.30) | 0.524 |

^a^ Values are odds ratios (95% confidence interval; p-value (* significant))

^b^ Odds ratio for the increase in 1 standard deviation of diet quality score

^c^ Q= quartile of diet quality score

^d^ Model 1 includes age, sex, energy intake, residence area, educational level, marital status, diabetes history, tobacco use and physical activity

^e^ Model 2 further includes baseline BMI

^f^ Model 3 further includes follow-up BMI

Abbreviations

rMED = Relative Mediterranean Diet

ModMDS = Modified Mediterranean Diet Score

DASH = Dietary Approaches to Stop Hypertension

HPDI = Healthful Plant-based Diet Index

WHO-HDI = World Health Organization Healthy Diet Indicator

Ptrend = p-value for linear trend across quartiles

**Table S10. Associations from multivariable Cox proportional hazards models between diet quality scores (continuous and quartiles) and depression diagnosis from primary care data in Analytical sample 2 (N=4789)**

| **rMED** | Continuous^a,b^ | Q1^a,c^ | Q2^a,c^ | Q3^a,c^ | Q4^a,c^ | P_trend_ |
| --- | --- | --- | --- | --- | --- | --- |
| N cases / N total | - | 43 / 753 | 65 / 1143 | 75 / 1330 | 78 / 1563 |  |
| Person-years | - | 8 945.85 | 13 460.73 | 15 789.75 | 18 514.87 |  |
| Model 1^d^ | 0.91 (0.80; 1.03) | 1.00 (Reference) | 0.97 (0.66; 1.44) | 0.88 (0.60; 1.29) | 0.78 (0.53; 1.15) | 0.140 |
| Model 2^e^ | 0.91 (0.80; 1.03) | 1.00 (Reference) | 0.98 (0.66; 1.44) | 0.88 (0.60; 1.29) | 0.78 (0.53; 1.15) | 0.139 |
|  | | | | | |  |
| **ModMDS** | Continuous^a,b^ | Q1^a,c^ | Q2^a,c^ | Q3^a,c^ | Q4^a,c^ |  |
| N cases / N total | - | 51 / 992 | 56 / 1108 | 74 / 1264 | 80 / 1425 |  |
| Person-years | - | 11 775.64 | 13 103.1 | 14 924.82 | 16 907.58 |  |
| Model 1^d^ | 0.96 (0.84; 1.09) | 1.00 (Reference) | 0.91 (0.62; 1.34) | 1.05 (0.73; 1.52) | 0.94 (0.65; 1.38) | 0.950 |
| Model 2^e^ | 0.96 (0.84; 1.10) | 1.00 (Reference) | 0.92 (0.63; 1.35) | 1.06 (0.73; 1.53) | 0.95 (0.65; 1.38) | 0.959 |
|  | | | | | |  |
| **DASH** | Continuous^a,b^ | Q1^a,c^ | Q2^a,c^ | Q3^a,c^ | Q4^a,c^ |  |
| N cases / N total | - | 62 / 1126 | 55 / 1090 | 56 / 1098 | 88 / 1475 |  |
| Person-years | - | 13 268.1 | 12 863.66 | 13 014.39 | 17 565.01 |  |
| Model 1^d^ | 0.91 (0.79; 1.05) | 1.00 (Reference) | 0.84 (0.58; 1.21) | 0.77 (0.53; 1.11) | 0.84 (0.59; 1.18) | 0.341 |
| Model 2^e^ | 0.91 (0.79; 1.05) | 1.00 (Reference) | 0.84 (0.58; 1.21) | 0.76 (0.52; 1.10) | 0.83 (0.59; 1.17) | 0.319 |
|  | | | | | |  |
| **HPDI** | Continuous^a,b^ | Q1^a,c^ | Q2^a,c^ | Q3^a,c^ | Q4^a,c^ |  |
| N cases / N total | - | 66 / 1142 | 47 / 1134 | 73 / 1192 | 75 / 1321 |  |
| Person-years | - | 13 433.68 | 13 460.13 | 14 108.68 | 15 708.67 |  |
| Model 1^d^ | 0.96 (0.83; 1.10) | 1.00 (Reference) | 0.64 (0.44; 0.93) | 0.89 (0.63; 1.26) | 0.81 (0.57; 1.17) | 0.648 |
| Model 2^e^ | 0.96 (0.83; 1.10) | 1.00 (Reference) | 0.63 (0.43; 0.92) | 0.88 (0.62; 1.24) | 0.81 (0.57; 1.16) | 0.630 |
|  | | | | | |  |
| **WHO-HDI** | Continuous^a,b^ | Q1^a,c^ | Q2^a,c^ | Q3^a,c^ | Q4^a,c^ |  |
| N cases / N total | - | 55 / 795 | 45 / 948 | 83 / 1522 | 78 / 1524 |  |
| Person-years | - | 9 370.22 | 11 214.23 | 18 059.05 | 18 067.65 |  |
| Model 1^d^ | 0.81 (0.70; 0.94) | 1.00 (Reference) | 0.57 (0.38; 0.85) | 0.58 (0.39; 0.85) | 0.49 (0.33; 0.73) | 0.003* |
| Model 2^e^ | 0.81 (0.70; 0.93) | 1.00 (Reference) | 0.57 (0.38; 0.85) | 0.57 (0.39; 0.84) | 0.48 (0.32; 0.72) | 0.002* |

^a^ Values are hazard ratios: (95% confidence interval; p-value (* significant))

^b^ Hazard ratios for the increase in 1 standard deviation of diet quality score

^c^ Q= quartile of diet quality score

^d^ Model 1 includes age, sex, energy intake, residence area, educational level, diabetes history, tobacco use and physical activity

^e^ Model 2 further includes baseline BMI

Abbreviations

rMED = Relative Mediterranean Diet

ModMDS = Modified Mediterranean Diet Score

DASH = Dietary Approaches to Stop Hypertension

HPDI = Healthful Plant-based Diet Index

WHO-HDI = World Health Organization Healthy Diet Indicator

Ptrend = p-value for linear trend across quartile

**Text S1. Patient Health Questionnaire (PHQ-9)**

Over the last 2 weeks, how often have you been bothered by any of the following problems?

|  | Not at all | Several days | More than half the days | Nearly every day |
| --- | --- | --- | --- | --- |
| 1. Little interest or pleasure in doing things |  |  |  |  |
| 1. Feeling down, depressed, or hopeless |  |  |  |  |
| 1. Trouble falling or staying asleep, or sleeping too much |  |  |  |  |
| 1. Feeling tired or having little energy |  |  |  |  |
| 1. Poor appetite or overeating |  |  |  |  |
| 1. Feeling bad about yourself, or that you are a failure or have let yourself or your family down |  |  |  |  |
| 1. Trouble concentrating on things, such as reading the newspaper or watching television |  |  |  |  |
| 1. Moving or speaking so slowly that other people could have noticed? Or the opposite, being so fidgety or restless that you have been moving around a lot more than usual |  |  |  |  |
| 1. Thoughts that you would be better off dead or of hurting yourself in some way |  |  |  |  |
